# Monthly Record of Deaths in the City of Buffalo

**Published:** 1858-07

**Authors:** H. D. Garvin

**Affiliations:** Health Physician


ART. IX.—*Report of Mortality in Buffalo for the month of May,* 1858.
By H. D. GARVIN, M. D., Health Physician.

| DISEASES. | No. | Males. | Females. | No Sex given. |
|---|---|---|---|---|
| Accidentally Killed, | 2 | 2 | | |
| Albuminuria, | 1 | | 1 | |
| Apoplexy, | 2 | | 2 | |
| Asthma, | 1 | | 1 | |
| Atrophia, | 1 | | 1 | |
| Born Sick, | 2 | 2 | | |
| Bronchitis, | 2 | 1 | 1 | |
| Chin Cough, | 1 | | 1 | |
| Congestion of Lungs, | 2 | 2 | | |
| Convulsions, | 9 | 7 | 2 | |
| Croup, | 1 | 1 | | |
| Dropsy, | 2 | 1 | 1 | |
| Drowning, | 5 | 4 | 1 | |
| Delirium Tremens, | 2 | 2 | | |
| Fever, Typhoid, | 7 | 4 | 3 | |
| " Scarlet, | 3 | 2 | 1 | |
| " Puerperal, | 2 | | 2 | |
| Gastro Enteritis, | 1 | 1 | | |
| Heart Disease, | 1 | | 1 | |
| Hæmorrhage | 3 | 1 | 2 | |
| Hydrocephalus, | 7 | 7 | | |
| Intemperance, | 2 | 1 | 1 | |
| Measles, | 1 | 1 | | |
| Morbus Cerebis, | 2 | 1 | 1 | |
| Marasmus, | 3 | 3 | | |
| Old Age, | 3 | 1 | 2 | |
| Phthisis Pulmonalis, | 16 | 7 | 9 | |
| Peritonitis, | 1 | 1 | | |
| Paralysis of Lungs, | 1 | | | 1 |
| Pneumonia, | 11 | 7 | 4 | |
| Still Born, | 5 | 2 | 3 | |
| Tabes Mesenterica, | 1 | 1 | | |
| Teething, | 1 | 1 | | |
| Typhoid Pneumonia, | 3 | 2 | 1 | |
| Whooping Cough, | 3 | 2 | 1 | |
| Total, | 111 | | | |

SEXES.

| | |
|---|---|
| Males, | 67 |
| Females, | 41 |
| Sex not given, | 3 |
| Total, | 111 |

AGES.

| | | | |
|---|---|---|---|
| Still-born, | 5 | Between 20 years and 30 years, | 10 |
| 1 day, | 0 | " 30 " " 40 " | 14 |
| 1 day and 30 days, | 6 | " 40 " " 50 " | 5 |
| Between 1 month and 6 months, | 8 | " 50 " " 60 " | 4 |
| " 6 months and 12 " | 9 | " 60 " " 70 " | 2 |
| " 1 year " 3 years, | 21 | " 70 " " 80 " | 2 |
| " 3 " " 5 " | 11 | " 80 " " 90 " | 1 |
| " 5 " " 10 " | 5 | " 90 " " 100 " | 0 |
| " 10 " " 20 " | 8 | " 100 " | 0 |
| | 73 | | 38 |
| Ages not given, | 0 | | 111 |
| Total, | 111 | | |

NATIVITIES.

| | | | |
|---|---|---|---|
| American, | 76 | Prussian, | 0 |
| German, | 19 | Italy, | 0 |
| Irish, | 10 | French, | 2 |
| English, | 3 | Scotch, | 1 |
| Canadian, | 0 | Bohemia, | 0 |
| Holland, | 0 | Country not given, | 0 |
| Total, | | | 111 |

